# Soluble glucocorticoid-induced tumor necrosis factor receptor regulates Helios expression in myasthenia gravis

**DOI:** 10.1186/s12967-019-1916-1

**Published:** 2019-05-22

**Authors:** Yi Li, Shumei Yang, Zhibin Li, Huanyu Meng, Wanling Jin, Huan Yang, Weifan Yin

**Affiliations:** 1 Department of Neurology, Xiangya Hospital, Central South University, 87 Xiangya Road, Changsha, Hunan China; 2 Department of Neurology, Second Xiangya Hospital, Central South University, 137 People Road, Changsha, Hunan China

**Keywords:** GITRL, GITR, Helios, Myasthenia gravis, Regulatory T cells

## Abstract

**Background:**

Helios is important for functional and phenotype stability of regulatory T cells (Tregs). However, the role of Helios in autoimmune diseases and its regulation remains unclear. This study aimed to investigate the role of Helios^+^ Tregs in myasthenia gravis (MG) and glucocorticoid-induced tumor necrosis factor receptor (GITR) and its ligand (GITRL) in the modulation of Helios.

**Method:**

Multicolor flow cytometry was performed to analyze Helios^+^ Tregs in peripheral blood from MG patients and healthy donors (HDs). Enzyme-linked immunosorbent assay (ELISA) was used to determine the levels of soluble GITRL/GITR in plasma. Tregs were isolated via magnetic separation and treated with recombinant GITRL and GITR-Fc. Membrane GITRL on Tregs and expression of Helios and other markers (FOXP3, CD25, CD39, CTLA-4, PD-L1 and IL-10) involved in immunosuppressive activity were determined by flow cytometry.

**Result:**

Both Helios^+^ Tregs and soluble GITR were decreased in generalized MG (GMG) patients (n = 14), compared with HDs (n = 14) and ocular MG (OMG) patients (n = 16). Helios^+^ Tregs possessed greater immunosuppressive capacity compared to Helios^−^ Tregs. Further analysis indicates soluble GITR was negatively correlated with quantitative MG score and promoted Helios expression and enhanced function of Tregs independently of membrane GITRL.

**Conclusion:**

This work demonstrates abnormal changes in Helios^+^ Tregs and soluble GITR in MG, as well as direct regulation of Helios by GITR in the context of Tregs. This work provides new insight into the role of GITR in the regulatory pathway of Helios and pathogenesis of MG.

## Background

Myasthenia gravis (MG) is T cell-dependent autoimmune disorder caused by antibodies targeting the neuromuscular junction, and affects 40–180 per million people with an annual incidence of 4–12 per million people [1]. It has been widely accepted that imbalance between T helper cells and regulatory T cells (Tregs) plays an essential role in the pathogenesis of MG. Abnormalities in the quantity or function of Tregs have been demonstrated in multiple studies of MG [2–4]. Investigating the key factors that modulate the function or phenotypic stability of Tregs is important for understanding the mechanism of MG and developing new therapeutic strategies.

Helios, a member of the Ikaros family of zinc finger proteins, was once thought to be a marker of thymic Tregs that were differentiated from peripheral Tregs [5, 6]. However, recent studies have suggested that Helios is involved in Treg function and phenotype stability. Helios expression in Tregs ensures a suppressive and anergic phenotype in intense inflammatory responses [7]. Overexpression of Helios enhances Treg function in cooperation with FOXP3 [7]. Selective Helios deficiency within Tregs led to induction of an unstable phenotype and conversion of Tregs into T effector cells within the tumor microenvironment and was associated with increased production of proinflammatory cytokines [8]. Helios deficiency in Tregs also led to reduced expansion of pathogenic T cells, T follicular helper cell and Th1 effector cell responses, and T follicular regulatory cell function [9]. However, the role of Helios^+^ Tregs in autoimmune diseases is still unclear, and little is known about its regulatory pathway.

The glucocorticoid-induced tumor necrosis factor receptor (GITR, TNFRSF18) and its ligand glucocorticoid-induced tumor necrosis factor receptor ligand (GITRL, TNFSF18) are members of the tumor necrosis factor/tumor necrosis factor receptor (TNF/TNFR) superfamily. GITR is expressed on various types of immune cells and constitutively expressed at a high level in Tregs [9]. GITRL is found on antigen-presenting cells, including dendritic cells, macrophages [10, 11]. GITRL/GITR has been reported to participate in the development of immune response against tumors and infectious agents, as well as in autoimmune and inflammatory diseases [12]. The role of GITRL in modulating T cell response is unclear, but most studies support that GITRL promotes the immune response and enhances tumor rejection [11, 13, 14]; however, GITR exerts anti-inflammatory effects and decreases the autoimmune response [12]. The underlying mechanisms of these responses remain unclear. A previous study suggested that triggering GITR with its antibody correlated with a drastic loss of Helios [15]. Thus, we suggest that GITRL/GITR may exert their function partially through the regulation of Helios in Tregs.

Therefore, we investigated the profile of Helios expressed on Tregs in MG and the immunosuppressive potential of Helios^+^ Tregs versus Helios- Tregs. Further, we demonstrated that soluble GITR and GITRL (sGITRL/sGITR) facilitate Helios-independent membrane GITRL.

## Methods and materials

### Patients and samples

Patients with possible MG consulting at the Neurology Department of Xiangya Hospital, Changsha, China, and healthy donors (HDs) were recruited from June 2017 to April 2018. Diagnostic criteria were based on fluctuating muscle weakness, positive neostigmine test, and abnormal repetitive nerve stimulation test (RNS). The enrolled subjects were screened for not having undergone any prior immune suppression therapy or thymectomy, and were also determined to be not suffering from any other autoimmune diseases or infection.

Clinical assessments were performed by neurologists in the MG Diagnosis and Treatment Center, Xiangya Hospital. Patients were instructed to not take any acetylcholine esterase inhibitor within 12 h before examination. During the examination, 5 mL blood samples were collected. Ocular MG (OMG) or generalized MG (GMG) was determined according to the Myasthenia Gravis Foundation of America (MGFA) clinical classification. The quantitative MG (QMG) score was performed when the patients were enrolled and treated by glucocorticoid 8–12 weeks (oral prednisolone, beginning with 20 mg/day and gradually increasing to the target dose of 1 mg/kg/day).

Patients with crisis or impending crisis treated with lymphoplasmapheresis were also selected, and their exchanged blood was used to isolate regulatory T cells. Isolated regulatory T cells were cultured for further drug treatment and flow cytometry. The levels of nicotinic acetylcholine receptor antibody (AchR-Ab) or muscle-specific tyrosine kinase antibody (MuSK-Ab) were acquired from the DAAN Clinical Laboratory Central Company (Guangzhou, China). Level out-weight of 0.45 nMol was considered to be positive for AchR-Ab and 0.05 nMol for MuSK-Ab.

All patients and HDs signed informed consent forms. The study was approved by the Ethics Review Committee of Xiangya Hospital.

### Enzyme-linked immunosorbent assay (ELISA)

sGITR and sGITRL levels in plasma were determined by ELISA according to manufacturer’s instructions. Levels of sGITRL were measured via a pre-coated GITRL Human ELISA Kit (Invitrogen, Carlsbad, CA, USA). Levels of sGITR were measured via a Sandwich ELISA Kit (RayBiotech Inc, Norcross, GA, USA).

### Flow cytometry

Flow cytometry was performed on a FACS-Canto II system (BD Biosciences, San Jose, CA, USA). Blood mononuclear cells (PBMCs) or isolated Tregs were analyzed for surface expression or intracellular markers using the following anti-human antibodies: PE-Cy7-labeled anti-CD4 (clone:SK3, BD Biosciences), Percp-Cy5.5-labeled anti-CD25 (clone: M-A251, BD Biosciences), APC-Cy7-labeled anti-CD39 (clone: A1, BD Biosciences), BV421-labeled anti-CTLA4 (clone: BNI3, BioLegend, San Diego, USA), PE-labeled anti-PD-L1 (clone: 29E2A3, BioLegend), Alexa Fluor 647-labeled anti-FOXP3 (clone: 259D/C7, BD Biosciences), Alexa Fluor 488-labeled anti-Helios (clone: 22F6, BD Biosciences), PE-labeled IL-10(clone: JES3-19F1, BioLegend) and PE-labeled anti-GITRL(clone: REA841, Miltenyi Biotec, Bergisch Gladbach, Germany). For surface marker staining, cells were incubated with the antibodies for 30 min at 4 °C shielded from light, then washed with PBS. For intracellular transcription factor and IL-10 staining, cells were first permeabilized using the Cytofix/Cytoperm Fixation/Permeabilization Kit (BD Biosciences), and then cells were incubated with intracellular transfactor antibody in eBiosciences Staining Buffer (Thermo Fisher Scientific, Waltham, MA, USA) for 45 min at 4 °C shielded from light. For IL-10 staining, cells were stimulated by 5 μg/ml anti-CD3 monoclonal antibodies (clone: UCHT1, Merck-Millipore, Burlington, MA, USA) and 1 μg/ml anti-CD28 monoclonal antibodies (clone: 28.2, Biolegend) for 48 h and Brefeldin A Solution (5 μg/ml) was added at final 6 h before harvest. After staining, cells were washed and fixed with 1% paraformaldehyde. The LIVE/DEAD Fixable Dead Cell Stain Kit (Invitrogen) was used for exclusion of dead cells. The positivity for all markers was established based on fluorescence minus one (FMO) and isotypic controls. All data were analyzed by FlowJo software VX.

### PBMC and regulatory T cell isolation

The peripheral blood samples collected in tubes with EDTA were centrifuged to obtain supernatant plasma, then diluted 1:1 with sterile PBS at room temperature (18–25 °C) and centrifuged on lymphocyte isolation agent (TBD, Tianjin, China) at 900*g* for 30 min. The isolated PBMCs were collected and washed with PBS. Tregs were isolated from the PBMCs of the patients who had accepted lymphoplasmapheresis therapy using magnetic separation (Miltenyi Biotec), according to the manufacturer’s instructions.

### Cell culture and drug treatment

Isolated Tregs were seeded in a 96-well round-bottom plate at 2 × 10^5^ cells per pore. Plates were coated overnight (12–16 h) with 10 μg/mL anti-CD3 mAb (clone: UCHT1, Merck-Millipore) before adding the cells. Cells were cultured in RPMI 1640 supplemented with Gibco 10% heat inactivated fetal bovine serum (Thermo Fisher Scientific), 1% sodium pyruvate (Thermo Fisher Scientific), 10,000 U/mL penicillin, 100 mg/mL streptomycin and 2 mM l-glutamine(Thermo Fisher Scientific). To investigate the effect of sGITRL/sGITR, Tregs were incubated with 0.2 µg/mL, 1 µg/mL, or 5 µg/mL GITRL (Novus Biologicals, Centennial, CO, USA) or 0.4 µg/mL, 2 µg/mL, or 10 µg/mL GITR-Fc refusion protein (BioLegend) for 72 h. Heat-inactivated GITRL (5 µg/mL) or human IgG (10 µg/mL, Dingguo, Beijing, China) were used as controls. Then, 10^5^ cells were collected and analyzed by flow cytometry.

### Statistical analysis

Data are expressed as mean ± SEM or median. The distribution of each parameter was evaluated by the Kolmogorov–Smirnov test. For data with normal distribution and homogeneity of variance, unpaired or paired Student’s *t*-test or one-way analysis of variance (ANOVA) was used. Tukey test was used for multiple comparison between different groups and post linear trend test was used to analyze the tendency. The Wilcoxon signed-rank test or Kruskal–Wallis test was used to compare data with a non-normal distribution, and Dunn’s test was used for multiple comparison. Correlations were assessed by Spearman’s rank correlation coefficients. A p-value < 0.05 was considered significant. All statistical analyses were performed using GraphPad Prism software (version 5.0).

## Results

### Frequencies of Helios^+^ Tregs in GMG patients decreased and were correlated to QMG scores

**Fig. 1:**
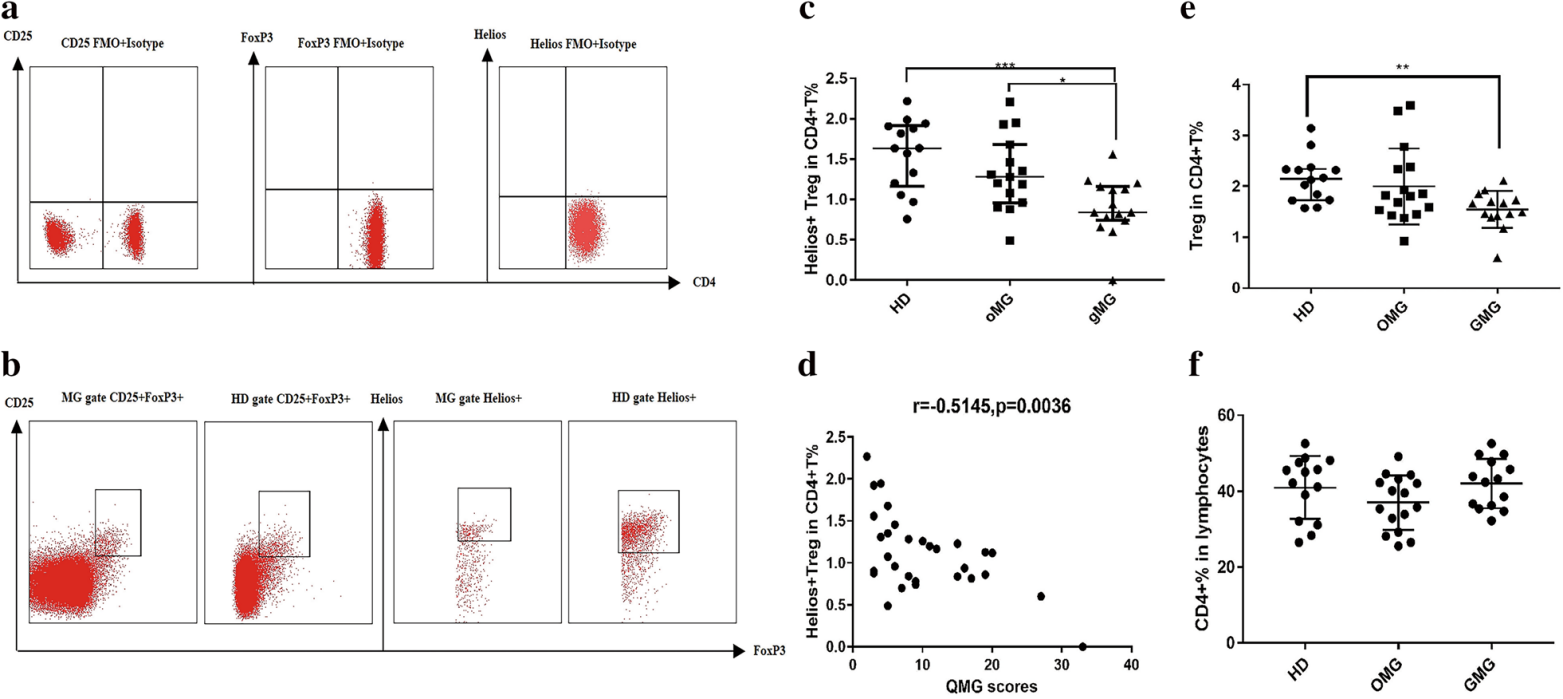
Frequencies of Helios^+^ Tregs in MG patients compared to HDs. **a** The FMO and isotype controls are shown. **b** Analysis of CD25, FOXP3, and Helios expression among CD4^+^ T cells in representative peripheral blood samples from HDs and patients with MG. **c** Frequencies of Helios^+^ Tregs among CD4^+^ T cells in HDs (n = 14), and patients with OMG (n = 14), and GMG (n = 16) (median/interquartile range values). **d** Correlation between frequencies of Helios^+^ Tregs among CD4^+^ T cells in MG patients with their QMG scores. **e**, **f** Frequency of Tregs in GMG patients decreased compared to HDs (*p *= 0.0059) and there was no different of CD4^+^ T frequency in each groups (*p *= 0.1435) (*p < 0.05, **p < 0.01, ***p < 0.005, ****p < 0.001)

A total of 55 patients suspected to have MG were reviewed, and 41 were confirmed to be MG cases. Among these cases, 6 were concomitant autoimmune thyroiditis, 1 was concomitant pemphigus; 2 patients were infected with hepatitis B virus, 2 patients were suffering with tuberculosis, and 11 subjects were excluded. In total, 30 MG patients and 14 HDs were enrolled. Patient information is provided in Table 1. There was no difference in age or sex ratio among each group. First, we analyzed the frequencies of Helios^+^ Treg in peripheral blood from the OMG and GMG patients and HDs by flow cytometry. We gated in CD4^+^ CD25^+^ FOXP3^+^ and Helios^+^ to distinguish Helios^+^ Tregs in PBMCs (Fig. 1a, b). It was found that frequencies of Helios^+^ Tregs in GMG patients were significantly lower than those in HDs (median values 0.84% vs. 1.63%, *p *= 0.0009) and OMG patients (median values 0.84% vs. 1.28%, *p *= 0.0402), while there was no difference between OMG patients and HDs (median values 1.28% vs. 1.63%, *p *= 0.6914). We also found that the frequency of Helios^+^ Tregs were negatively correlated with QMG score in MG patients (r = -0.5145, *p *= 0.0036, Fig. 1c, d). The frequency of Tregs in GMG patients were also found decreased compared to HDs (*p *= 0.0059, Fig. 1e) and there was no different of CD4^+^T frequency in each groups (*p *= 0.1435, Fig. 1f). No correlation was found between QMG scores and frequency of Tregs (*p *= 0.1646, not shown).

**Table 1:** Summary of clinical characters of enrolled subjects and healthy donors

Groups	OMG	GMG	HD	p
Number of cases	16	14	14	
Age (mean ± SD)	36.2 ± 16.7	44.29 ± 9.21	39.14 ± 10.9	0.238
Sex (male/female)	9/7	6/8	6/8	0.6935
QMG score (mean ± SD)	5.36 ± 1.84	16.14 ± 7.37		
MGFA I	16	0		
MGFA II	0	5		
MGFA III	0	6		
MGFA IV	0	2		
MGFA V	0	1		
AchR-Ab positive	14	13		
MuSK-Ab positive	0	1		
Seronegtive	2	0		
Thymoma	0	1		

No significant differences were observed in age and gender ratio among three groups (p > 0.05)

*OMG* ocular myasthenia gravis, *GMG* generalized myasthenia gravis, *HD* healthy donors, *MGFA* Myasthenia Gravis Foundation of America Clinical Classification, *AchR-Ab* nicotinic acetylcholine receptor antibody, *MuSK-Ab* muscle specific tyrosine kinase antibody

### Glucocorticoid treatment increased frequency of Helios^+^ Tregs in MG patients

**Fig. 2:**
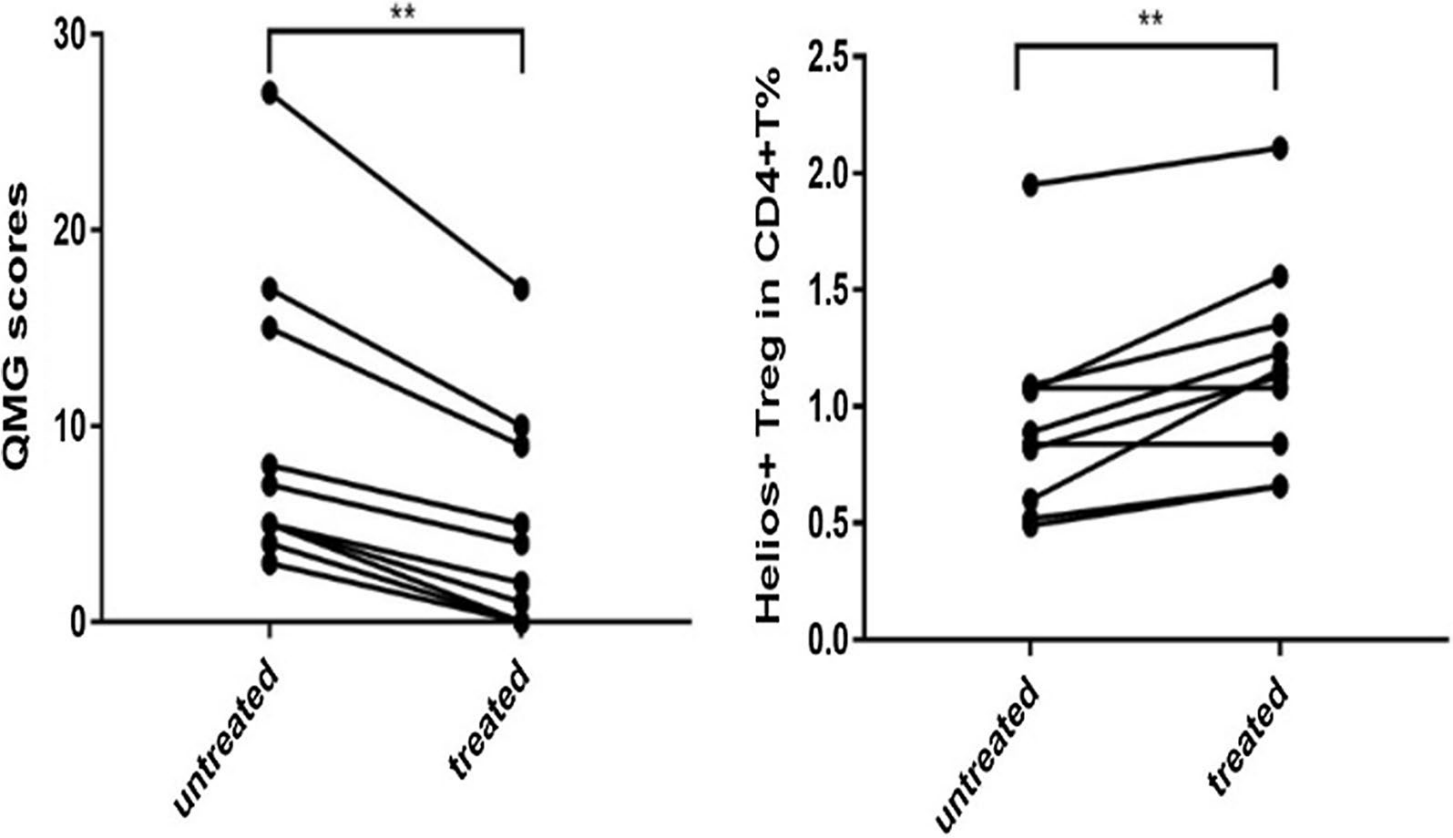
Change of QMG scores and frequency of Helios^+^ Tregs after glucocorticoid therapy in MG patients (n = 10). Although the QMG scores significantly decreased after therapy (*p *= 0.0039), frequency of Helios^+^ Tregs showed no difference (*p *= 0.3125). (*p < 0.05, **p < 0.01, ***p < 0.005, ****p < 0.001)

In order to analyze if changes in Helios^+^ Tregs were linked to treatment response, we performed longitudinal analysis of QMG and frequency of Helios^+^ Tregs. In 10 MG patients who received 8–12 weeks oral prednisolone therapy, the QMG scores were dramatically decreased (*p *= 0.0039); accompanying with the frequency of Helios^+^ Tregs increased (p = 0.0078) (Fig. 2).

### Helios^+^ Tregs expressed higher level of CD39 and FOXP3

**Fig. 3:**
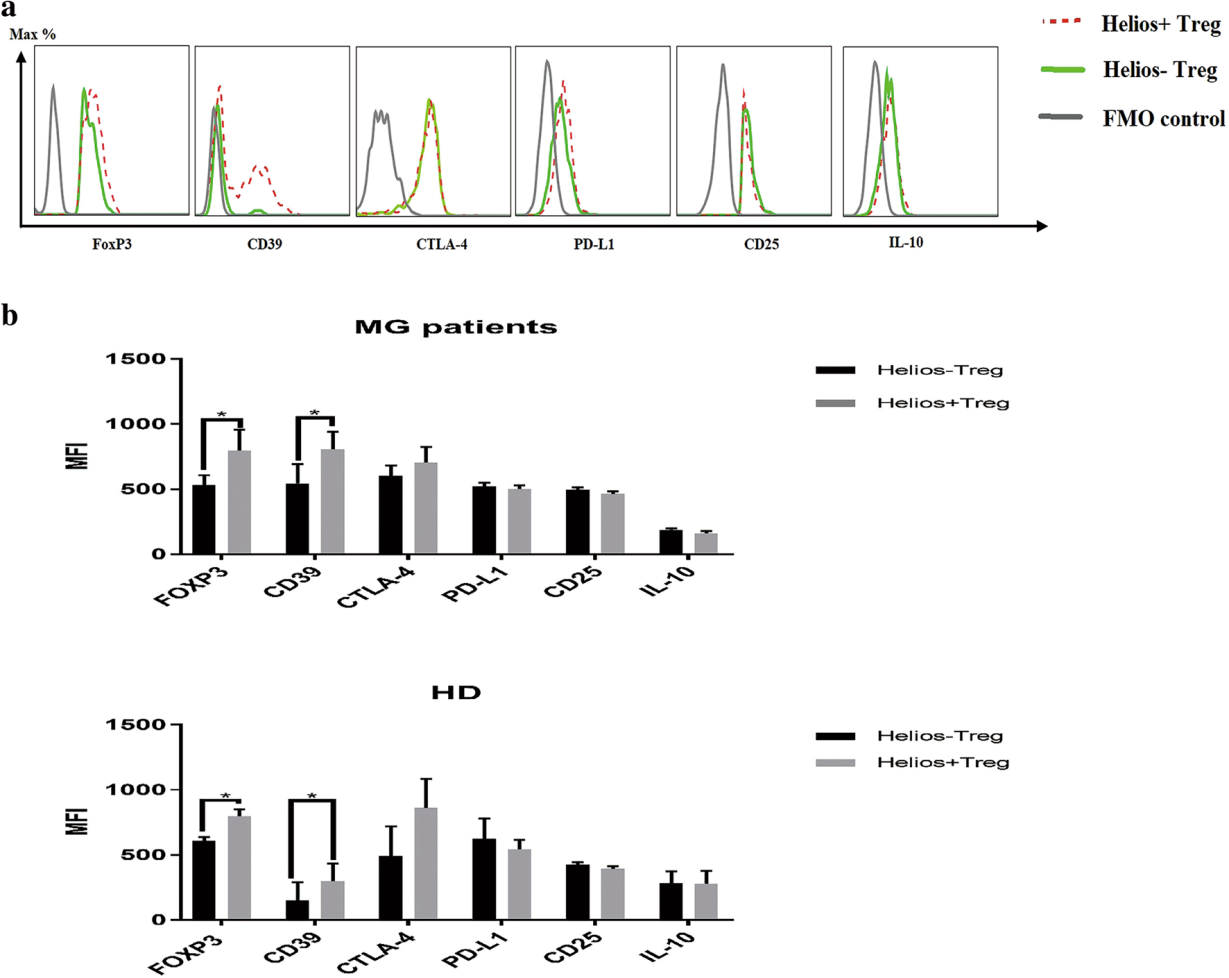
Analysis of suppressive function-related molecules in Helios^+^ Tregs and Helios^−^ Tregs. **a** Histogram showing the representative comparison of FOXP3, CD39, CTLA-4, PD-L1, CD25 and IL-10 expression in Helios^+^ Tregs and Helios^−^ Tregs. **b** Helios^+^ Tregs expressed higher levels of FOXP3 and CD39 (*p *= 0.033, *p *= 0.0415, respectively) in MG patients and HDs. (*p < 0.05, **p < 0.01, ***p < 0.005, ****p < 0.001)

We next compared the expression of suppressive function-related molecules between Helios^+^ Tregs and Helios^−^ Tregs. An additional 10 MG patients and 10 HDs were recruited (Table 2). Blood samples were collected and analyzed by flow cytometry. We first gated CD4^+^CD25^+^FOXP3^+^ to distinguish Tregs. Then FOXP3, CD25, CD39, CTLA-4, PD-L1 and IL-10 were compared, respectively in Helios^+^ subgroup and Helios^−^ subgroup in these identified Tregs. The analysis shows that expression levels of FOXP3 and CD39 were higher in Helios^+^ Tregs than those in Helios^−^ Tregs. On the other hand, the levels of the other markers related to immunosuppressive activity, such as CD25, CTLA-4, PD-L1 and IL-10 were comparable between Helios^+^ Tregs and Helios^−^ Tregs, as shown in Fig. 3. Taken together, these results suggest that Helios^+^ Tregs may be more suppressive than Helios^−^ Tregs.

**Table 2:** Details of 10 patients and 10 heathy donors

Groups	MG	HD
Age (mean ± SD)	32.9 ± 13.3	36.4 ± 9.1
Sex (male/female)	5/5	4/6
QMG score(mean ± SD)	10.3 ± 5.4	
MGFA I	3	
MGFA II	6	
MGFA III	1	
AchR-Ab positive	9	
MuSK-Ab positive	0	
Seronegtive	1	
Thymoma	0	

*MG* myasthenia gravis, *HD* healthy donors, *MGFA* Myasthenia Gravis Foundation of America Clinical Classification, *QMG score* quantitative myasthenia gravis score, *AchR-Ab* nicotinic acetylcholine receptor antibody, *MuSK-Ab* muscle specific tyrosine kinase antibody

### sGITR were decreased in GMG patients and correlated to frequency of Helios^+^ Tregs

**Fig. 4:**
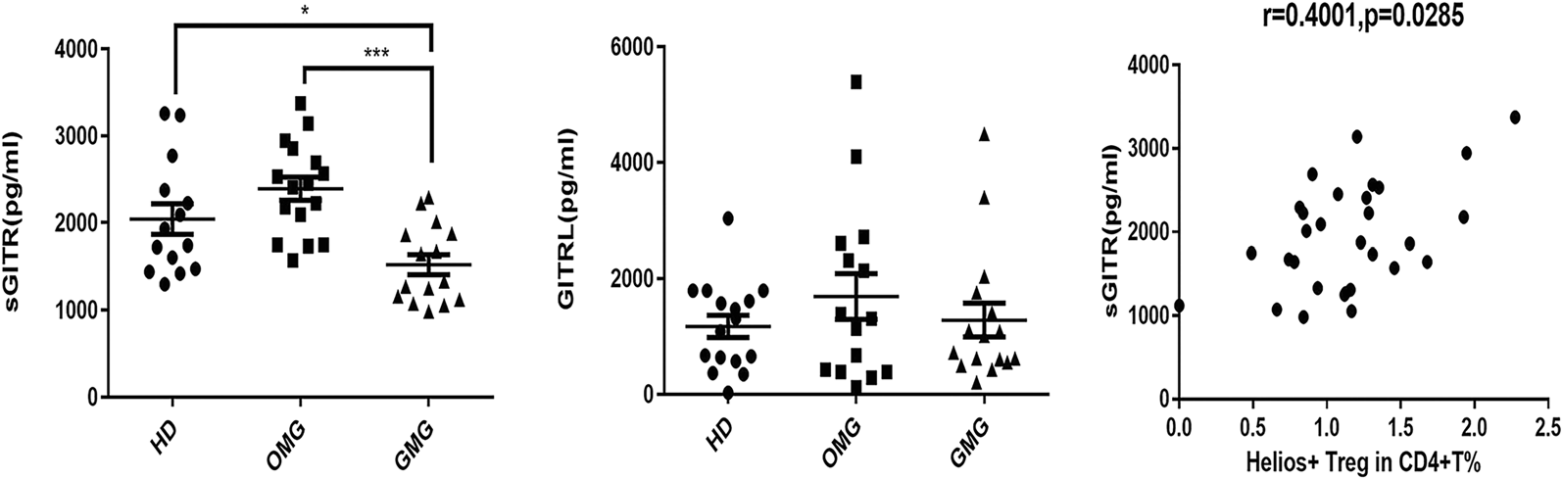
Level of soluble GITR and soluble GITRL in peripheral plasma. Soluble GITR in GMG patients was significantly lower than in HDs (*p *= 0.0377) and OMG patients (*p *= 0.0002). For soluble GITRL, there was no significant difference among HDs, OMG patients, and GMG patients (*p* = 0.8250). There was a positive correlation between soluble GITR and frequency of Helios^+^ regulatory T cells (*r* = 0.4001, *p *= 0.0285). (*p < 0.05, **p < 0.01, ***p < 0.005, ****p < 0.001)

Previous studies have revealed that Treg-mediated suppression of the immune response is abrogated by triggering GITR [13, 16] and high levels of sGITRL or sGITR in serum have been reported in autoimmune diseases, such as rheumatoid arthritis (RA), Hashimoto’s thyroiditis, systemic lupus erythematosus (SLE), and Sjögren’s syndrome (SS) [17–20]. In this study, we determined the levels of sGITRL and sGITR in the plasma of MG patients and HDs by ELISA. Our study found that sGITRL showed no difference among all groups (median values 1201 pg/mL vs. 1309 pg/mL vs. 867.9 pg/mL, *p *= 0.8250). However, plasma levels of sGITR in GMG were significantly lower than those in HDs (mean = 1522 ± 115 pg/mL vs. 2043 ± 175.4 pg/mL *p *= 0.0002) and OMG patients (mean = 1522 ± 115 pg/ml vs. 2392 ± 133.7 pg/ml, *p *= 0.0287), with no difference between OMG patients and HDs (mean = 2392 ± 133.7 pg/ml vs. 2043 ± 175.4 pg/ml, *p *= 0.10) (Fig. 4). A Spearman correlation analysis was also performed to investigate the relationships between the levels of sGITR and Helios^+^ Treg frequency. We found that sGITR positively correlated with Helios^+^ Treg frequency (r = 0.4001, *p *= 0.0285).

### GITR directly promoted Helios expression and function in Tregs in vitro

**Fig. 5:**
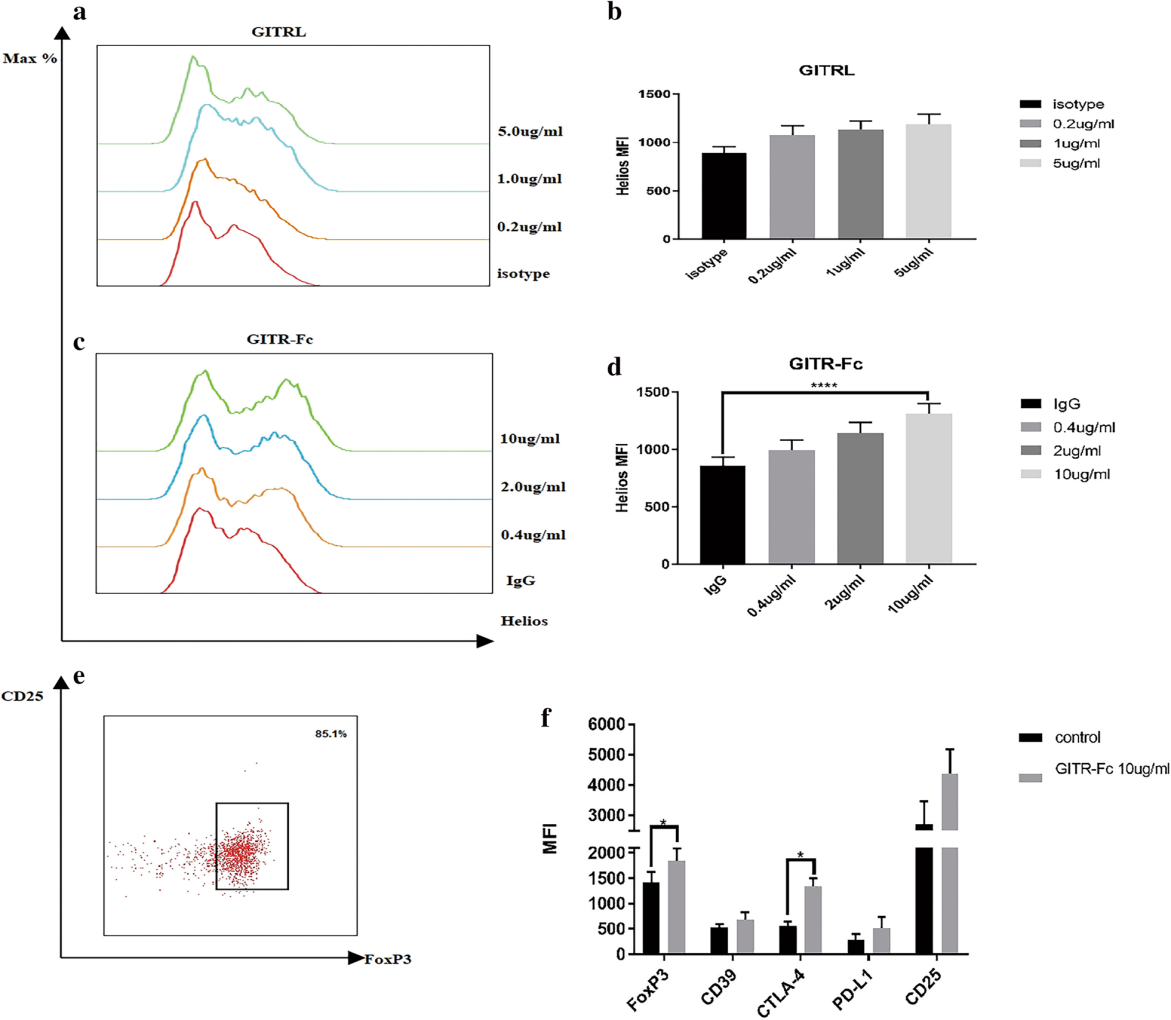
GITR and GITRL directly promoted Helios expression and function in Tregs in vitro. **a**, **b** Recombinant GITRL had a modest effect on promoting Helios expression, although there is no statistical significance. **c**, **d** GITR-Fc promoted Helios expression in Tregs (ANOVA, *p *< 0.0001) in a dose-dependent manner (post linear trend test, *p *< 0.0001). **e** Purity of Tregs after isolation by magnetic separation. **f** Expression of FOXP3 and CTLA-4 increased (*p *= 0.0219, p = 0.002, respectively) after treatment with GITR-Fc (10 µg/mL). (*p < 0.05, **p < 0.01, ***p < 0.005, ****p < 0.001)

Our previous data indicated that GITR was associated with frequency of Helios^+^ Tregs and severity of MG. This observation suggests that GITRL and GITR modulate Helios expression and further affect suppressive function-related molecules expression in Tregs. To test this hypothesis, we first investigated the direct effect of GITRL and GITR in Tregs. PBMCs from 5 eligible MG patients that received lymphoplasmapheresis therapy were collected to isolate Tregs by magnetic separation. Details of the patients are listed in Table 3, and the purity of CD4^+^CD25^+^FOXP3^+^ Tregs is demonstrated in Fig. 5e. The purified Tregs were cultured and treated with GITRL (0.2 µg/mL, 1 µg/mL, 5 µg/mL) or GITR-Fc (0.4 µg/mL, 2 µg/mL, 10 µg/mL) for 72 h. Cells treated with Heat-inactivated GITRL (5 µg/mL) or human IgG (10 µg/mL) or none of them were used as controls. Flow cytometry was performed to analyze the expression of Helios. We found that GITRL had no significant effect in promoting Helios expression. However, GITR-Fc promoted Helios expression level (*p *< 0.0001), and this upregulation was in a dose-dependent manner (post linear trend test, *p *< 0.0001), as shown in Fig. 5a–d. Then, the expression of FOXP3, CD39, CTLA-4, and PD-L1 in Tregs was analyzed after treatment with GITR-Fc (10 µg/mL). Both FOXP3 and CTLA-4 expression were increased (Fig. 5f).

**Table 3:** Details of patients accepted lymphoplasmapheresis

No.	Age (years)	Gender	QMG score	MGFA classification	Antibody status	Thymoma	MV
1	48	Female	19	IV	AchR-Ab(+)	No	No
2	32	Male	33	IV	AchR-Ab(+)	No	No
3	57	Male	32	IV	AchR-Ab (+)	No	No
4	23	Female	29	IV	AchR-Ab(+)	No	No
5	47	Male	22	V	MuSK-Ab(+)	No	Yes

*MGFA Classification* Myasthenia Gravis Foundation of America Clinical Classification, *QMG score* quantitative myasthenia gravis score, *AchR-Ab* nicotinic acetylcholine receptor antibody, *MuSK-Ab* muscle specific tyrosine kinase antibody, *MV* mechanical ventilation

### GITR regulated Helios expression independent of membrane GITRL in Tregs

**Fig. 6:**
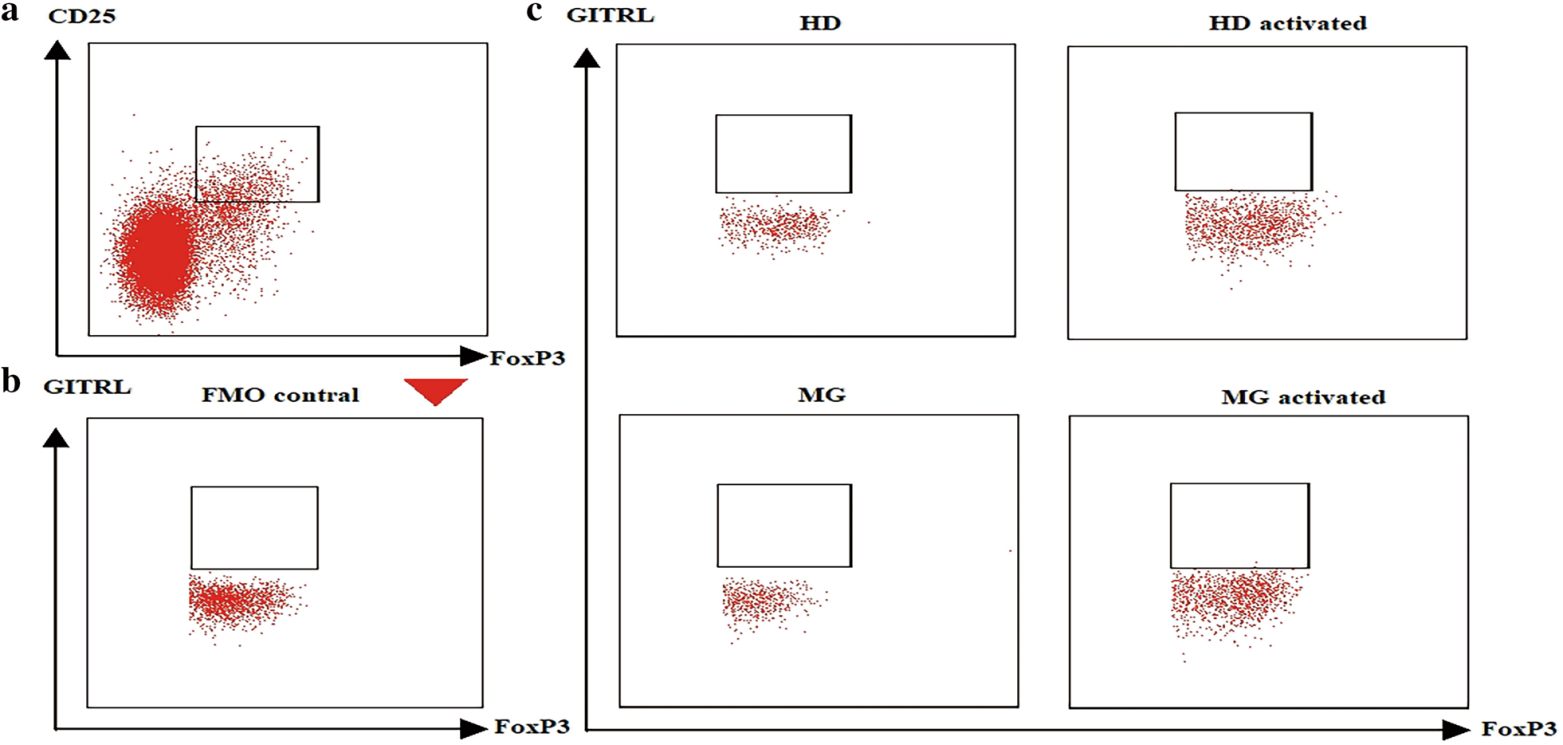
sGITR regulated Helios-independent membrane GITRL in Tregs. **a** Tregs were gated from CD4^+^ cells. **b** FMO and isotype controls and gating strategy are shown. **c** Analysis of GITRL expression shows that GITRL was not expressed on the surface of Tregs, even when activated by pre-coating with anti-CD3 mAbs in both HDs (n = 4) and MG patients (n = 3)

Although we demonstrate that sGITR regulates Helios expression, the underlying mechanism remains unclear. Considering that reverse signaling through membrane GITRL after engagement by sGITR is involved in the inflammatory response in macrophages and dendritic cells [21, 22], we speculated that sGITR may function in Tregs in a similar way. Thus, we investigated GITRL expression on the surface of Tregs. Flow cytometry was used to detect membrane GITRL and FMO, and isotype was used to establish positivity. We found that Tregs, both in MG patients (n = 3) and HDs (n = 3), did not express membrane GITRL, even when activated by pre-coating with anti-CD3 mAb, as shown in Fig. 6.

## Discussion

In this study, we show that both frequency of Helios^+^ Tregs and level of sGITR are decreased in GMG patients, as compared to HDs and OMG patients, and are correlated with the severity of MG. Our results also indicate that GITR, not GITRL, promoted expression of Helios and enhanced the modulatory function of Tregs in MG. In addition, we found that GITR may regulate Helios expression independent of membrane GITRL.

We demonstrate an abnormal decrease of Helios^+^ Tregs in MG. It is known that human Tregs comprise a specific subpopulation of T cells, which play an essential role in immune homeostasis. Many autoimmune diseases are associated with homeostatic imbalance of regulatory and conventional effector T cells. However, a unique marker to define Tregs is still lacking. Previously defined markers for the identification of Tregs in MG patients, including CD25, FOXP3, and GITR [3, 4], are also increased during effector T cell activation; and a potent suppressive subset of Tregs cannot be characterized by these markers. To analyze the contribution of human Tregs to the pathogenesis of MG or any other autoimmune disease, it is necessary to have reliable criteria to accurately determine functional Tregs. Helios, an Ikaros transcription family member, has been recently suggested to be a marker for naturally occurring Tregs. It has become evident that Helios^+^ Tregs, in contrast to Helios^−^ Tregs, have enhanced suppressive potential [23], migratory potential into inflamed tissues, and possess a highly demethylated Treg-specific demethylated region (TSDR) in the *FOXP3* gene, suggesting a more stable phenotype. Helios may therefore define a subset of Tregs with a putative role in mediating self-tolerance. Xu et al. also found an abnormal decrease of Helios^+^ Tregs in MG [4]. However, in other autoimmune diseases, Alexander et al. and Takatori et al. reported that Helios^+^ Tregs were expanded in active SLE but unaltered in RA [7, 24], These conflicting reports indicate the lack of knowledge regarding the functionality and the role of Helios-expressing Tregs in autoimmune diseases. The present study investigated Helios expression in Tregs from patients with MG. We found that frequencies of Helios^+^ Tregs were significantly decreased in GMG patients compared to HDs and OMG patients, and were negatively correlated with disease severity. Further analysis of markers involved in immunosuppressive activity shows that Helios expressing-Tregs expressed higher level of CD39 and FOXP3. These observations suggest that abnormal reduction of Helios^+^ Tregs may contribute to the pathophysiology and development of MG. The comparable frequencies of Helios^+^ Tregs between OMG patients and HDs may be due to our limited sample size (14 HDs and 16 OMG patients).

GITRL and GITR are members of the TNF/TNFR superfamily. Their contribution to immune system regulation is complex and important, as demonstrated by experimental models of autoimmunity, inflammation, and tumors. Abnormal elevated levels of GITRL or/and GITR in serum have been reported in SLE, RA, Sjögren’s syndrome, and Hashimoto’s thyroiditis, which all correlated with disease severity or level of autoantibody [17–20]. However, to our knowledge, few studies have focused on the immunological roles of soluble GITRL/GITR involved in MG. In this study, we found levels of GITRL in plasma were comparable in HDs, OMG, and GMG patients while GITR levels in GMG patients were significantly lower than in both HDs and OMG patients, and negatively correlated with QMG scores. Further analysis indicated that GITR was associated with the frequency of Helios^+^ Tregs. The association between Helios^+^ Tregs and soluble GITR drew our attention to the regulatory effect of GITRL/GITR.

How GITRL and GITR modulate immune reaction remains unclear. Previous research implies that GITRL induces immune response and inhibits activity of Tregs, while Kim et al. showed that GITRL signal is context-dependent, having stimulatory effects on effector T cells and inhibitory effects through Tregs [15]. Our observation indicates that human GITRL did not impair Tregs function [15]. The modulatory effect of GITR-Fc has been observed previously. Nocentini et al. and Galuppo et al. noticed anti-inflammatory effects of GITR-Fc [25, 26]; however, the underlying mechanism was unknown. Galuppo et al. demonstrated that GITR-Fc reduced inflammation similar to the genetic inhibition of GITR expression in mice and they speculated that GITR-Fc exerts its effect depending on the neutralization of GITRL, similar to the effect of anti-mGITRL Ab [12]. However, our results show that GITR-Fc may ameliorate immune response/inflammation by directly enhancing the function of Tregs rather than neutralization of GITRL. We also detected expression of membrane GITRL on Tregs, further indicating that GITR affecting Treg function. In contrast to macrophages or dendritic cells which affected by GITR through membrane GITRL reverse signaling, GITR exert its function independent of membrane GITRL [21, 22]. The signaling pathway of GITR in the context of Tregs remains unknown; however, we speculate that there may exist unknown GITR receptor on Tregs. More studies are required to confirm this hypothesis.

This study has several limitations. First, the limit sample size hindered us to discriminate some small difference, such as whether the frequency of Helios^+^ Tregs differed in OMG patients and HDs. Second, we could not directly compare suppressive activity of Helios^+^/Helios^−^ Tregs through co-culture with effector T cells as Helios is a nuclear transcription factor. Third, the purity of Tregs isolated by MACS was not satisfactory and effect of non-Treg cells cannot be completely excluded. It may be improved by fluorescence activated cell sorting.

## Conclusion

In conclusion, our data show that both frequency of Helios^+^ Tregs and level of sGITR are decreased in GMG, compared to HDs and OMG, and correlate with severity of MG. Our data also indicate that GITR directly regulates expression of Helios in MG. This work provides new insight into the regulatory pathway of Helios by GITR and the pathogenesis of MG.

## Data Availability

The datasets used and/or analyzed during the current study are available from the corresponding author on reasonable request.

## Abbreviations

MG: myasthenia gravis
Tregs: regulatory T cells
GITR: glucocorticoid-induced tumor necrosis factor receptor
GITRL: glucocorticoid-induced tumor necrosis factor receptor ligand
TNF/TNFR: tumor necrosis factor/tumor necrosis factor receptor
RNS: repetitive nerve stimulation
HD: healthy donors
sGITR/sGITRL: soluble GITR/GITRL
OMG: ocular MG
GMG: generalized MG
MGFA: Myasthenia Gravis Foundation of America
QMG score: quantitative myasthenia gravis score
AchR-Ab: nicotinic acetylcholine receptor antibody
MuSK-Ab: muscle specific tyrosine kinase antibody
PBMCs: peripheral blood mononuclear cells
FMO: fluorescence minus one
PBS: phosphate buffer saline
RA: rheumatoid arthritis
SLE: systemic lupus erythematosus
SS: Sjögren’s syndrome
TSDR: Treg specific demethylated region
